# Metabolomics Identifies Biomarker Signatures to Differentiate Pancreatic Cancer from Type 2 Diabetes Mellitus in Early Diagnosis

**DOI:** 10.1155/2021/9990768

**Published:** 2021-11-25

**Authors:** Hongmin Xu, Lei Zhang, Hua Kang, Jie Liu, Jiandong Zhang, Jie Zhao, Shuye Liu

**Affiliations:** Department of Clinical Laboratory, The Third Central Hospital of Tianjin, Tianjin Institute of Hepatobiliary Disease, Tianjin Key Laboratory of Extracorporeal Life Support for Critical Diseases, Artificial Cell Engineering Technology Research Center, No. 83, Jintang Road, Hedong District, Tianjin 300170, China

## Abstract

**Methods:**

Plasma metabolic profiles in 26 PC patients, 27 DM patients, and 23 healthy volunteers were examined using an ultraperformance liquid chromatography coupled with tandem mass spectrometry platform. Differential metabolite ions were then identified using the principal component analysis (PCA) model and the orthogonal partial least-squares discrimination analysis (OPLS-DA) model. The diagnosis performance of metabolite biomarkers was validated by logistic regression models.

**Results:**

We established a PCA model (R2X = 23.5%, *Q*2 = 8.21%) and an OPLS-DA model (R2X = 70.0%, R2Y = 84.9%, *Q*2 = 69.7%). LysoPC (16 : 0), catelaidic acid, cerebronic acid, nonadecanetriol, and asparaginyl-histidine were found to identify PC, with a sensitivity of 89% and a specificity of 91%. Besides, lysoPC (16 : 0), lysoPC (16 : 1), lysoPC (22 : 6), and lysoPC (20 : 3) were found to differentiate PC from DM, with higher accuracy (68% versus 55%) and higher AUC values (72% versus 63%) than those of CA19-9. The diagnostic performance of metabolite biomarkers was finally validated by logistic regression models.

**Conclusion:**

We succeeded in screening differential metabolite ions among PC and DM patients and healthy individuals, thus providing a preliminary basis for screening the biomarkers for the early diagnosis of PC.

## 1. Introduction

Pancreatic cancer (PC) is a highly malignant gastrointestinal tumor characterized by rapid progression and early metastasis, which results in an incurability rate of 96% and a recurrence rate of 80% after diagnosis [1, 2]. Malignancies are projected to become the 2nd leading cause of cancer-related death by 2030 [3, 4]. Surgical resection is considered as the only potential curative treatment for PC patients. However, more than 80% of PC patients at the time of diagnosis already have had an unresectable, locally advanced, and metastatic tumor [5]. Consequently, PC has a 5-year survival rate of approximately 5%, which is the lowest among all types of malignancies [6]. Early diagnosis has been accepted to hold promise for improving the prognosis of PC.

Carbohydrate antigen 19-9 (CA19-9), the only Food and Drug Administration (FDA)-approved blood-based biomarker, is considered as a common tumor marker to phenotype PC. Unfortunately, CA19-9 is nonspecific, thus being widely accepted as a poor screening tool for PC diagnosis [6, 7]. The low application value of CA19-9 may result from its little expression in Lewis-negative patients who account for about 10% of the total number of PC patients [8]. Besides, previous studies have reported that the sensitivity and specificity of CA19-9 in distinguishing cancer from chronic pancreatitis is commonly no better than 65% or 60%, respectively [8, 9]. The complex relationships among chronic pancreatitis, diabetes mellitus (DM), and PC hinder the clinical application of CA 19-9 in the early diagnosis of PC. Chronic pancreatitis is an inflammatory disease that causes pancreatic inflammation and fibrotic injury. Chronic pancreatitis is a risk factor for both PC and DM because PC and DM patients often suffer from endocrine pancreatic dysfunction [10]. About 80% of patients with chronic pancreatitis will develop overt DM, which is an independent risk factor for mortality in patients with chronic pancreatitis [10–12]. In addition, CA19-9 is strongly correlated with crucial biochemical markers of metabolic compensation in diabetic patients [10]. The elevated CA19-9 in diabetic patients is reported to result from chronic pancreatitis instead of PC [10]. These findings suggest that individuals with a high level of CA 19-9 in early diagnosis may simply have developed DM instead of PC. CA19-9-induced misdiagnosis can increase the psychological burden on patients and delay the treatment. Therefore, it is urgent to explore alternative noninvasive biomarkers for distinguishing PC from type 2 DM in early diagnosis.

Metabolomics is a powerful approach to identify cancer-related biomarkers. This technology has demonstrated its feasibility in identifying metabolite alterations in various types of tumors, including PC, recently [8]. The advantages of metabolomic technology include the following: (1) high sensitivity: gas chromatography-mass spectrometry (GC-MS) and liquid chromatography-mass spectrometry (LC–MS), the main techniques used in metabolomics analysis, can detect low concentration metabolites, which is useful for early cancer diagnosis due to subtle metabolic changes in early cancer [6]; (2) high throughput: the metabolomic platform is efficient to analyze multiple samples at the same time; and (3) small sample size: a study recruiting up to 30 patients per group has suggested that metabolomics is useful in PC detection [6]. These advantages of metabolomics ensure that the identified biomarkers are reliable for further clinical tests.

Therefore, the current study aims to employ metabolomics technology to identify biomarkers that can differentiate PC and DM in early diagnosis. To fulfill the design, this study combined ultrahigh-performance liquid chromatography (UPLC) and high-resolution mass spectrometry (HRMS) with the high resolution and high-quality precision in metabolomics analysis. Plasma metabolic profiles were examined to demonstrate the differences in metabolites among PC and DM patients and healthy volunteers. This study offers potential biomarkers for distinguishing PC from DM in early diagnosis and provides a theoretical basis for an indepth investigation on mechanisms behind the development of the two diseases.

## 2. Materials and Methods

### 2.1. Study Subjects

This study was approved by the Medical Ethics Committee of the Tianjin Third Central Hospital, China (approval number: IRB2017-013-01). All subjects and/or their guardians signed informed consent forms.

#### 2.1.1. Pancreatic Cancer Patients

26 patients who were diagnosed with PC by B-ultrasound, computed tomography, magnetic resonance imaging, and endoscopy and admitted to the Third Central Hospital of Tianjin between May 2018 and March 2019 were enrolled in the pancreatic cancer group (PC group). The diagnosis was performed in accordance with the diagnostic criteria established by the Chinese consensus on diagnosis and treatment of PC (2014 version). Patients were excluded from participation if they had a history of receiving surgery, other tumors, and viral hepatitis infection.

#### 2.1.2. Diabetic Patients

The diagnosis of type 2 DM met the diagnostic criteria developed by the American Diabetes Association (ADA) in 2010. Patients with type 2 DM who received medical checkup and hospitalized in the endocrinology department of the Third Central Hospital of Tianjin over the same period were enrolled in the diabetes group (DM group). Subjects were excluded from participation if they had a history of malignancy, pancreatic injury, and viral hepatitis infection. A total of 27 subjects were enrolled in the DM group.

#### 2.1.3. Healthy Volunteers

Volunteers were excluded from participation if they had a history of diabetes, pancreatic injury, and viral hepatitis infection. A total of 23 healthy volunteers with normal liver function and renal function were selected as healthy controls (normal group).

#### 2.1.4. Collection of the Demographic Data

Clinical assessments such as age, sex, body mass index (BMI), and underlying diseases were characterized for the study population. Fasting plasma glucose (GLU) and glycosylated hemoglobin (HbAlc) were analyzed using a Roche Modular P automatic biochemical analyzer. CA19-9 was measured with a Roche Cobas 8000 automatic biochemical analyzer.

#### 2.1.5. Collection of Plasma Specimens

Analyzed serum samples were obtained from three different cohorts of participants in PC group, DM group, and normal group. To reduce the effects of food fluctuations on metabolism, enrolled subjects were required to have a light diet, avoiding seafood, spicy food, smoking, and drinking the day before the sample collection. In the early morning of the next day, 3 mL of fasting venous blood was collected and placed in a purple vacuum blood collection tube (BD Biosciences, CA, USA), with ethylenediaminetetraacetate (EDTA) as an anticoagulant. Then, the blood was centrifuged at 3500 rpm for 10 min at 4°C. Next, the upper plasma was collected and stored in an ultralow temperature freezer at −80°C until analysis. All the blood samples and clinical data were collected with the informed consent of the subjects. This study was authorized by the Clinical Research Ethics Committee of the Third Central Hospital of Tianjin.

### 2.2. Experimental Methods

#### 2.2.1. Plasma Sample Pretreatment

Prior to the analysis, the plasma samples were thawed at room temperature. 100 *μ*L of plasma was mixed with 300 *μ*L of methanol. The mixtures were shaken vigorously for 30 s, followed by being placed at 4°C for 5 min. Then, the mixtures were centrifuged at 12000 ×g for 15 min at 4°C. The resultant supernatant was collected and passed through a 0.22 *μ*m filter. Next, the samples to be tested were obtained. Equal volumes of each sample were taken and mixed to prepare a quality control (QC) sample, which was then injected repeatedly to monitor the MS performance.

### 2.3. Liquid Chromatograph

The liquid chromatograph used in this study was an Accela Ultra High-Performance Liquid Chromatography (UPLC) System (Thermo Fisher Scientific MA), which was equipped with a binary solvent gradient elution system and an automated sample-loading system. Mobile phase A was a 0.1% formic acid aqueous solution, and mobile phase B was a 0.1% formic acid acetonitrile solution (the flow rate was 200 *μ*L/min). The initial condition of the mobile phase consisted of 95% *A* solution and 5% *B* solution during the first 2.5 min. During the subsequent 3.5 min of elution, the proportion of *A* solution was linearly reduced from 95% to 5%. Next, the proportion of *B* solution was linearly increased from 5% to 95% over 3 min. Finally, equilibrate the column with the mobile phase in the initial gradient ratio over 3 minutes.

#### 2.3.1. Mass Spectrometry (MS) Analysis

An LTQ Orbitrap XLTM combined mass spectrometer was used in this study for MS analysis. The acquisition was performed in positive ion mode. The mass calibration was performed with a calibration mixture consisting of caffeine, Ultramark 1621, and tetrapeptide MRFA. Samples were analyzed in a random order.

#### 2.3.2. Sample Quality Control

To evaluate the stability and repeatability of the UPLC/MS detection system (Thermo Fisher Inc.). A total of 20 QC samples were analyzed, and 10 QC samples were continuously tested before specimen analysis to examine the repeatability of the system. Then a QC injection was conducted for quality control, every 9^th^ sample. Samples (*n* = 61 in total) were analyzed in random order. Each sample (including the QC sample) was inserted into the blank after detection to avoid cross-contamination.

### 2.4. Statistical Analysis

#### 2.4.1. Data Preprocessing

The raw data acquired from the UPLC/MS detection platform was directly imported into MZmine2.0 software (free analysis software) for data preprocessing and normalization, including peak detection, alignment, and normalization (with the total ionic strength of each sample as a normalization factor). After the analysis, a (*i* × *j*) two-dimensional peak table was obtained. Each row (*i*) represented a sample, and each column (*j*) represented a metabolite variable, i.e., the m/z value represented the ion peak intensity (peak integral area). The 80% rule was used to remove variables with too many missing values, and then, the MZmine2.0 was employed to assign values to variables with few missing values.

MZmine 2.0 software was employed to extract ion chromatographic peak intensity with a signal-to-noise ratio (S/N) > 10, retention time (RT) shift <±0.2 min, and mass-to-charge ratio (m/z) deviation <±0.02. Then, peak identification, matching, and normalization were performed. The relative amount of plasma metabolites was expressed as the integrated area of the chromatographic peak.

#### 2.4.2. Multivariate Statistical Analysis

The data obtained from the preprocessing as described in Section 2.3.1 were imported into SIMCA-P +12.0.1.0 software (Umetrics, Sweden) for analysis. To visualize the differences in plasma metabolic profiles between groups, a multidimensional model was established. Firstly, the principal component analysis (PCA) model was developed and used as an unsupervised pattern recognition method to observe the overall distribution trend of samples and evaluate the stability of test results of the devices. The clustering was displayed in the PCA plot. And outliers were removed. Secondly, the orthogonal partial least-squares discriminant analysis (OPLS-DA) model was constructed to identify the overall difference in the metabolic spectrum between the two groups. Variables responsible for distinguishing different sample groups (differential metabolites) were selected based on the variable importance in the projection (VIP) values. Briefly, variables containing “0” in the confidence interval on VIP plots and coefficient plots were excluded from metabolites with VIP scores above 1.0. Next, loading plots were used to ensure that the selected metabolites have a large degree of change and high reliability.

The parameters for evaluating the quality of the OPLS-DA model were R2X, R2Y, and *Q*2. R2X and R2Y describe the percentage of *X* and Y matrix information that can be explained by the model, respectively. *Q*2 reveals the predictive ability of the model. R2 and *Q*2 with their values closer to 1 indicate the more stable and reliable model. Effective and reliable models should meet the following criteria: the model with *Q*2 > 50% was considered to be valid, while the model with *Q*2 > 90% was considered to be excellent.

#### 2.4.3. Single-Dimensional Statistical Analysis

For clinical data from each group, statistical analysis was performed using SPSS 22.0 software. Measurement data were expressed as *X* ± SD (standard deviation), *χ*2 test was used to compare the count data between the two groups. Differential metabolites were screened, and a nonparametric test (Mann–Whitney *U* test) was performed. The binary logistic regression analysis was applied to assess the influence of metabolites on the occurrence of diseases. The correlation between metabolite variation and the occurrence of diseases was evaluated by quartile agreement. Combinations of differential metabolites were identified by the one-way ANOVA and Tukey HSD tests. ROC curves were plotted to assess the diagnostic efficacy of different combinations. All statistical treatments were performed on both sides, and *P* < 0.05 was considered statistically significant.

#### 2.4.4. Screening and Identification of Differential Metabolites

Some characteristic ions were identified by comparing the standard chromatographic peak with the mass spectrum peak (including the first mass spectrum peak and the second fragment mass spectrum peak). The method to identify characteristic ions was as follows: (1) since the Orbitrap XL mass spectrum with a resolution of 100,000 (FMHW) was employed for the first-order mass spectrometry scan, the HMDM was searched for the exact mass-to-charge ratio (m/z) of the characteristic ions. The search results were verified by the database (http://hmDM.ca/). The instrument parameters were reset according to the selected variables, and the QC samples were subjected to secondary MS/MS scanning to obtain the characteristic secondary ion mass spectra. Next, an accurate mass-to-charge ratio (m/z) and second-order mass spectrum were used to search for mass in Frontier 6.0 and the HMDB database for obtaining the results via structure-based derivation.

#### 2.4.5. Clinical Performance Evaluation

To calculate the relative risk value for each metabolite, we conducted a logistic regression analysis on the metabolites selected from ANOVA and multiple comparisons between groups. We used a corrected *P* value threshold of 0.001 to account for the 21 metabolites analyzed as variables in the logistic regression analysis. First, the insulin resistance and secretion were log-transformed. The logistic regression models were then adjusted for age, gender, body mass index (BMI), fasting blood glucose, and log-transformed values. We analyzed metabolites as both continuous and categorical variables (with quartile values used as cutoff points) to estimate the odds ratio for each SD increment and each quartile increment. To classify patients based on their metabolic profiles, we constructed a logistic regression model using the *R* and glm function. Specificity, sensitivity, area under the curve (AUC), accuracy, and kappa values were obtained from a 5 times repeated 5-fold cross-validation. Top metabolites selected from one-way ANOVA analysis and Tukey's HSD test were used to construct different biomarker combinations in the training set. We further assessed the performance of each combination on the classification of individuals between groups.

## 3. Results

### 3.1. Demographic Data of the Study Subjects

The demographic data of the study subjects are summarized in Table 1.

### 3.2. Total Ion Current Map

**Figure 1:**
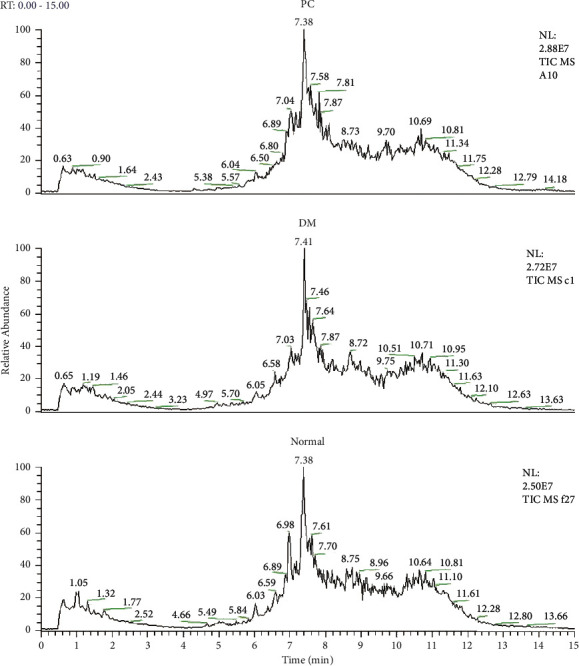
Total ion chromatogram (TIC) of plasma samples from pancreatic cancer (PC), diabetics (DM), and healthy controls (normal), indicating the PC- and DM-caused variation in the content of small molecule metabolites.

Samples were analyzed according to the chromatographic and mass spectrometry conditions, as described in Section 2. The total ion chromatograms (TIC) of plasma samples obtained from patients with pancreatic cancer (PC group), diabetes (DM group), and healthy controls (normal group) were shown in Figure 1. Figure 1 describes the differences in peak height and peak area under the same retention time among groups, which indicates the difference in relative content of the same substance among groups and the existence of disease. For example, when the retention time is 7.87 min, the spectrum of plasma in the PC group shows a distinct peak, while the DM group and the normal group show no significant change. In addition, significant differences in the peak heights and peak areas on the TIC maps are found among the three groups (i.e., PC, DM, and normal groups) within 6.03–7.41 min.

The observed differences in TIC of plasma samples might also exist among different samples (individuals) within the same group. The plasma metabolic profiles obtained by using UPLC/MS involve a lot of information. The determination of information of interest in the absence of target components (i.e., known biomarkers) is difficult. Therefore, a combination of multiple data analysis methods is required to screen potential metabolic markers and potential links among study subjects by integrating, classifying, and analyzing multidimensional and scattered data.

### 3.3. Results from PCA Analysis

**Figure 2:**
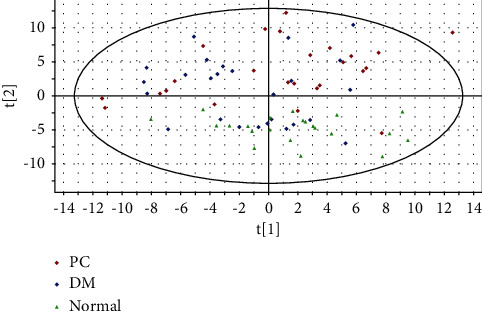
PCA scores of plasma metabolic profiles in PC, DM, and normal groups, suggesting good analytical stability and reliability of the UPLC-MS/MS workflow, as well as the plasma metabolite profile. *t*[1]: the first principal component; t[2]: the second principal component.

According to the results from data preprocessing, as described in Section 2.3.1, a total of 319 metabolite ions extracted were used for further analyses. The PCA analysis was first performed as an unsupervised learning method to evaluate the analytical stability and reliability. The score plot of the PCA analysis was shown in Figure 2. As shown in Figure 2, the two orthogonal axes (principal components), *t*[1] and *t*[2], used for modeling accounted for 23.5% and 8.21% of the variable information, respectively (R2X = 23.5% and *Q*2 = 8.21%). Besides, Figure 2 suggests the tendency of separation, with a few discrete points and insignificant dispersion (95% confidence interval). These findings indicate that the distribution state of the overall sample and instrument stability are good. However, there is an obvious separation tendency between the PC group and the normal group, suggesting the differences in plasma metabolic profiles of PC patients and healthy volunteers.

### 3.4. Results from Analysis Based on the OPLS-DA Model

**Figure 3:**
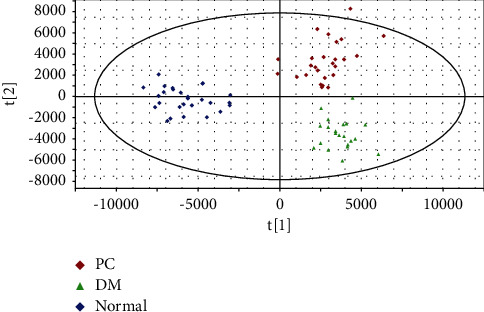
OPLS-DA scores of plasma metabolic profiles in PC, DM, and normal groups, demonstrating the good fitness and predictive ability of the model in identifying the differential metabolite ions. *t*[1]: the first predicted principal component; *t*[2]: the first orthogonal principal component.

**Figure 4:**
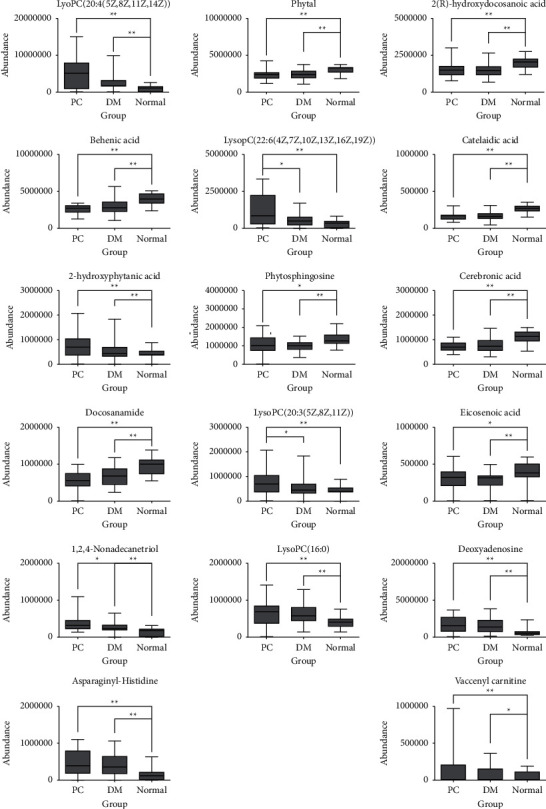
Differential metabolite ions in PC, DM, and normal groups. Mann–Whitney *U* test: ^*∗*^*P* 0.05; ^*∗∗*^ *P*0.01. These results suggest the importance of differential metabolite ions we screened and identified for differentiating PC and DM.

Differences in plasma metabolic profiles among PC patients, DM patients, and healthy volunteers were identified by the PCA analysis. Considering the complexity of interference factors, to further eliminate the influence of nondisease factors, maximize the separation, eliminate the influence of nondisease factors caused by the complexity of interference factors, maximize the separation and search for differential metabolites in plasma among the three groups, the OPLS-DA model was constructed to remove the information unrelated to the sample classification and distinguish plasma metabolic profiles among groups. The OPLS-DA score plots of the spectra demonstrate two predicted principal components and five orthogonal components, with the R2X, R2Y, and *Q*2 values being 70.0%, 84.9%, and 69.7%, respectively (Figure 3), suggesting good fitness and predictive ability. Besides, in Figure 4, a clear trend of clustering is observed among groups (PC, DM, and normal groups), indicating that the disease is the main factor of the clustering trend.

### 3.5. Identification of Differential Metabolites

The identification of metabolites was carried out as described in Section 2. A total of 17 kinds of differential metabolite ions were detected, among which phospholipids (lysophosphatidylcholine (lysoPC), sphingosine, and ceramide) account for the majority, while other ions (such as eicosanoids and long-chain fatty acids) are responsible for cell differentiation and energy metabolism. Results from the nonparametric test show significant differences in the content of 4 metabolite ions between the PC group and the DM group (Figure 4). LysoPC (22 : 6), lysoPC (20 : 3), and 1,2,4-nonadecanetriol are significantly higher in the PC group than those in the DM group and the normal group. Figure 4 also demonstrates that the content of lysoPC (16 : 0) in the PC group was significantly lower than that in the DM group. These results suggest the importance of lysoPC (22 : 6), lysoPC (20 : 3), 1,2,4-nonadecanetriol, and lysoPC (16 : 0) and their involved metabolic pathways for elucidating the relationship between the mechanisms of PC and DM, thus providing potential biomarkers for early diagnosis of PC.

Interestingly, the plasma metabolic profiles in the PC group were similar to those in the DM group. Compared to the normal group, the levels of lysoPC (20 : 4), deoxyadenosine, asparaginyl-histidine, and vaccenyl carnitine in the PC group and DM group were significantly increased, while the levels of phytal, 2 (R)-hydroxydocosanoic acid, behenic acid, catelaidic acid, 2-hydroxyphytanic acid, phytosphingosine, cerebronic acid, docosanamide, and eicosenoic acid in the PC group and DM group were significantly reduced (Figure 4).

### 3.6. Evaluation of Clinic Performance

Logistic regression models were used to assess the association between baseline metabolite levels and future disease occurrence after adjusting for age, sex, BMI, and fasting glucose. Most phospholipids, fatty alcohols, and peptides were found to have a stimulatory effect on the occurrence of PC and DM. Among the top metabolites selected in our study, a 0.381-fold, 0.286-fold, 1.099-fold, and 0.845-fold increase in the risk of DM occurrence (*P*=0.0051 to 0.0337) (Tables 2 and 3) for each increment of 1 SD in the log-transformed values of lysoPC (16 : 0), LysoPC (16 : 1), nonadecanetriol, and asparaginyl-histidine, respectively, was observed. Individuals with plasma metabolite levels in the top quartile had a 1.158- to 5.24-fold increased odds of developing DM compared to those in the lowest quartile. In addition, we also found a 1.441-fold, 3.026-fold, 1.915-fold, and 1.052-fold increase in the risk of PC occurrence (*P*=0.0011 to 0.0029) for each increment of 1 SD in the log-transformed values of lysoPC (22 : 0), lysoPC (22 : 3), nonadecanetriol, and asparaginyl-histidine, respectively, with a 2.475- to 5.241-fold odds between the top and lowest quartile. Beneficial effects on preventing incidence of PC and DM were observed in the metabolites of fatty acids and fatty amides groups (i.e., catelaidic acid, docosanamide, cerebronic acid, behenic acid). Catelaidic acid, docosanamide, cerebronic acid, and behenic acid were negatively correlated with the risk of DM occurrence, with odds ratios of 0.137–0.271. While catelaidic acid, cerebronic acid, and behenic acid were negatively correlated with the risk of PC occurrence, with odds ratios of 0.137–0.271. In the case of the distinction between PC and DM, only phospholipids (i.e., lysoPC (16 : 0), lysoPC (22 : 6), lysoPC (20 : 3), and lysoPC (16 : 1)) were detected with significant odds ratios. Among them, lysoPC (22 : 6) and lysoPC (20 : 3) were found to have a positive effect on the increased risk for future PC development in DM patients (odds ratio = 2.296 and odds ratio = 1.683, respectively). While lysoPC (16 : 0) and lysoPC (16 : 1) showed an opposite effect on the increased risk for future PC development in DM patients (odds ratio = 0.917 and odds ratio = 0.878, respectively) (Tables 2 and 3).

**Figure 5:**
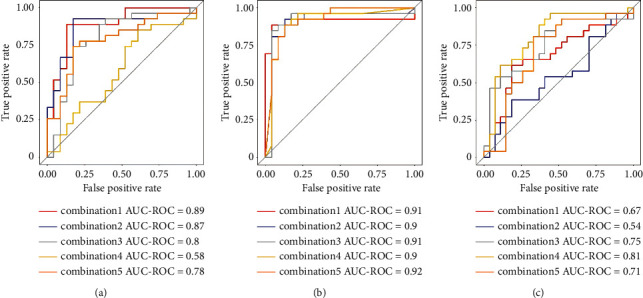
ROC curves of the metabolites (biomarker signature) results on plasma samples from all patients with pancreatic cancer versus healthy volunteers (a), from patients with diabetes mellitus versus healthy volunteers (b), and from patients with pancreatic cancer versus patients with diabetes mellitus (c). Models were tested through 5 repeated 5-fold cross-validation. AUC, area under the curve; combination1, model with top 5 metabolites selected from one-way ANOVA test; combination2, model with top 5 metabolites selected from Tukey's honest significance test between diabetes mellitus patients and healthy volunteers; combination3, model with top 5 metabolites selected from Tukey's honest significance test between pancreatic cancer patients and healthy volunteers; combination4, model with top 5 metabolites selected from Tukey's honest significance test between pancreatic cancer patients and diabetes mellitus patients; and combination5, model with all the metabolites involved in previous 4 models.

**Figure 6:**
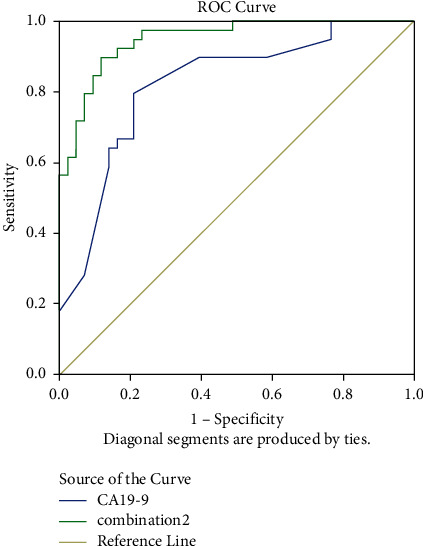
ROC curves of the diagnostic ability of combination2 versus CA19-9.

Five combinations were obtained through one-way ANOVA and Tukey's HSD test and were defined as combination1, combination2, combination3, combination4, and combination5, respectively, in this study. The combination1 involves differential metabolites (i.e., LysoPC (22 : 6), catelaidic acid, cerebronic acid, docosanamide, and asparaginyl-Histidine) that were screened through one-way ANOVA. The combination2 involves differential metabolites (i.e., lysoPC (16 : 0), catelaidic acid, cerebronic acid, nonadecanetriol, and asparaginyl-histidine) screened through Tukey's HSD (PC). The combination3 involves differential metabolites (i.e., lysoPC (22 : 6), catelaidic acid, cerebronic acid, docosanamide, and asparaginyl-histidine) screened through Tukey's HSD (DC). The combination 4 involves differential metabolites (i.e., lysoPC (16 : 0), lysoPC (16 : 1), lysoPC (22 : 6), and lysoPC (20 : 3)) screened through Tukey's HSD (PC). Combination5 consisted of all the metabolites involved in abovementioned four combinations. The combination2 demonstrated an outstanding performance in differentiating DM from the heathy population, with a diagnostic accuracy of 85.8% (95% CI: 82.8% to 88.9%), a kappa statistic of 71.3% (95% CI: 65.1% to 77.5%), an AUC of 0.95 (95% CI: 0.94 to 0.97), a sensitivity of 87.6% (95% CI: 83.9% to 91.3%), and a specificity of 87.6% (95% CI: 82.5% to 92.7%) (Figure 5 and Table 4). Besides, this combination also showed outstanding abilities in differentiating PC from healthy individuals, with a diagnostic accuracy of 88.6% (95% CI: 86.4% to 90.9%), a kappa statistic of 76.9% (95% CI: 72.2% to 81.7%), an AUC of 0.97 (95% CI: 0.96 to 0.99), a sensitivity of 89.0% (95% CI: 84.7% to 93.3%), and a specificity of 90.6% (95% CI: 86.1% to 95.1%) (Figure 5 and Table 4). In contrast, CA19-9 showed a poor ability in PC diagnosis, with a diagnostic accuracy of 81.3% (95% CI: 77.8% to 83.4%), a kappa statistic of 67.3% (95% CI: 60.3% to 74.7%), an AUC of 0.82 (95% CI: 0.76 to 0.87), a sensitivity of 79.1% (95% CI: 74.5% to82.6%), and a specificity of 82.6% (95% CI: 76.5% to 89.4%), as demonstrated in Figure 6 and Table 5.

The combination4 was found to demonstrate excellent diagnostic performance in differentiating PC from DM (Table 4). Figure 6 and Table 5 also show that the combination1–3 demonstrated higher ACU values than other combination did (0.904 vs. 0.872; 0.919 vs. 0.882; and 0.680 vs. 0.586, respectively). These findings suggest that the combined detection of metabolic substances can improve the diagnostic efficacy of PC and DM, thus indicating that the models shows stable and reasonable diagnostic performance in differentiating PC and DM from the healthy population.

## 4. Discussion

Early diagnosis is promising for improving the survival rates and prognosis of PC patients. However, the early diagnosis of PC is vulnerable to complex relationships among chronic pancreatitis, DM, and PC [10, 12]. Metabolites, influenced by the disease and identified by metabolomics, are accepted as the powerful biomarkers for discriminating between benign and malignant lesions [8]. The current study employed metabolomics technology to identify differential metabolites and found that biomarker signatures comprising of differential metabolites demonstrate good ability in distinguishing between PC and DM. The pancreas consists of two types of tissues, namely, exocrine and endocrine. Therefore, the pancreas has two different functions: an exocrine function that benefits digestion and an endocrine function that regulates blood sugar [13]. Hormones (such as insulin and glucagon) and any slight damage to the pancreas can lead to changes in metabolites [14]. The variations in blood plasma metabolic profiles can provide clues for identifying pancreatic diseases. For example, in 2011, Bathe et al. used a Proton Nuclear Magnetic Resonance (1H NMR)-based metabolomics approach to analyze 58 metabolites and succeeded in distinguishing malignant pancreatic lesions from hepatobiliary disease [15]. In the present study, the metabolomic profiles of plasma samples obtained from PC patients, DM patients, and healthy controls were identified and analyzed for the first time by using UPLC/MS-based metabolomics.

The feasibility of using metabolites as biomarkers for identifying PC has been reported previously [16–18]. In 2018, an untargeted and targeted metabolomics approach on 914 patients and 477 metabolites validated that multimarker signatures (nine metabolites and additionally, CA19-9) improved the diagnostic accuracy for detecting PC compared to CA19-9 alone [8]. The present study aims to identify more common and specific markers between PC and DM by analyzing the metabolite differences, provide an experimental basis for the indepth understanding of the two diseases from the perspective of pathogenesis, and provide guidance for the early diagnosis of PC. Therefore, optimized sensitivity and specificity of the assay were highlighted in this work. Under the condition that the PC incidence is between 0.71% and 0.85%, to reduce healthcare expenditure and improve patient survival, any new diagnostic test is required to meet the sensitivity of over 88% and the specificity of over 85% [8, 19]. Five biomarker signatures (i.e., lysoPC (16 : 0), catelaidic acid, cerebronic acid, nonadecanetriol, and asparaginyl-histidine) identified in this current study demonstrated a sensitivity of 89% and a specificity of 91%. These findings suggest a higher sensitivity and specificity than that of CA 19-9 reported previously (79% and 82%, respectively) [20]. Therefore, five biomarker signatures proposed in our study made significant progress in identifying PC. Additionally, the classifier including these five biomarker signatures demonstrated a high sensitivity and specificity of 88% in distinguishing patients with DM from healthy individuals in our study. The classifier showed a higher sensitivity (88% versus 59%) than the reported HbA1c and a specificity (88% versus 96%) similar to the reported HbA1c [21]. These findings suggest that the classifier including these five biomarker signatures allows for a more accurate exclusion of DM in individuals with a negative test result than HbA1c does.

The integrity of pancreatic acinar cells, production of digestive enzymes, and secretion of insulin depend on choline phospholipid metabolism. Therefore, phospholipids, including lysoPC, are significantly important for pancreatic cell function [22]. LysoPC is produced by lecithin cholesterol acyl transferase (LCAT) and phospholipase A2 (PLA2) enzyme-catalyzed hydrolysis of the fatty acid ester, which is responsible for cell proliferation, tumor cell infiltration, and inflammatory responses [23, 24]. Elevated plasma lysoPC levels were found in patients with type 2 DM and were reported to be associated with insulin resistance and chronic inflammation [25, 26]. Furthermore, mitochondrial oxidative stress in DM patients was documented to activate the protein kinase C (PKC) signaling pathway, which in turn increased the activity of PLA2 enzymes and promoted the production of lysoPC, thus increasing the serum levels of lysoPC [27]. In addition, studies have found the elevated activity of PLA2 enzymes in the pathological state of cancer, especially in advanced malignant tumors. These findings suggest an elevated lysoPC in the plasma of PC patients. Urayama et al. confirmed the significantly increased plasma levels of lysoPC in patients with PC by using an MS-based plasma metabolic profiling analysis [28]. Therefore, PC and DM show significantly increased lysoPC levels and disturbed phospholipid metabolic pathways. In our work, the biomarker signatures (i.e., lysoPC (16 : 0), catelaidic acid, cerebronic acid, nonadecanetriol, and asparaginyl-histidine) demonstrated sufficient ability to distinguish PC and DM. These findings might result from the similarly increased lysoPC levels and similarly disturbed phospholipid metabolic pathways in PD and DM. Of note, the biomarker signatures, comprised of phospholipids (i.e., lysoPC (16 : 0), lysoPC (16 : 1), lysoPC (22 : 6), and lysoPC (20 : 3)), showed increased accuracy (68% versus 55%) and AUC values (72% versus 63%) in identifying PC. Therefore, further studies on the underlying mechanisms of how phospholipids influence the occurrence of PC may contribute to developing a diagnostic test for the more accurate and earlier detection of PC.

Although the current study provides potential and promising biomarkers for differentiating PC from DM, it suffers from the limitation of small sample size. The validity and applicability of these markers needs to be further confirmed by data from a multicenter, large-sample clinical epidemiologic investigation. Besides, DM is accepted as a risk factor for PC and also a paraneoplastic syndrome caused by PC. In view of the potential contribution of DM to the early diagnosis of PC, a multicenter, large sample clinical epidemiologic investigation contributes to the establishment of a prospective study cohort of new-onset diabetes in PC and a screening system for the early diagnosis of pancreatic cancer. The screening system should also be developed in combination with the current biological indicators of early diagnosis of PC.

Taken together, the present study successfully identified 16 metabolite ions (i.e., lysoPC (20 : 4), lysoPC (22 : 6), lysoPC (20 : 3), deoxyadenosine, asparaginyl-histidine, vaccenyl carnitine, phytal, 2 (R)-hydroxydocosanoic acid, behenic acid, catelaidic acid, 2-hydroxyphytanic acid, phytosphingosine, cerebronic acid, docosanamide, eicosenoic acid, and 1,2,4-nonadecanetriol) that differ between PC patients and health controls. Meanwhile, there were 4 metabolite ions (i.e., lysoPC (16 : 0), lysoPC (16 : 1), lysoPC (22 : 6), and lysoPC (20 : 3)) that were found to demonstrate excellent abilities in differentiating PC from DM. Our study, therefore, provides potential biomarkers for differentiating PC from DM and also elucidates the relationship between PC and DM in terms of pathogenesis.

## Figures and Tables

**Table 1:** Demographic data of subjects.

Characteristic	PC group (*n* = 26)	DM group (*n* = 27)	Normal group (*n* = 23)
Sex			
Male	14	14	12
Female	12	13	11
Age (years, mean ± SD)	64.74 ± 7.93∗	56.93 ± 9.93	60.22 ± 6.65
Underlying diseases			
Acute coronary disease	2^#^	1	0
Hypertension	1	1	1
Cerebral infarction	1	1	0
Location			
Head of pancreas	15		
Body of pancreas	8		
Tail of pancreas	4		
BMI (mean ± SD)	19.32 ± 4.11^#^∗	22.98 ± 3.79	23.13 ± 3.62
Duration of DM (Mon, mean ± SD)	10.97 ± 7.66∗	14.26 ± 8.54	
Fasting plasma glucose (GLU, mmoL/L, mean ± SD)	7.32 ± 1.09^#^	7.65 ± 1.43^#^	5.11 ± 0.63
Glycosylated hemoglobin (HbAlc,%, median, range)	8.9 (6.3, 11.5)^#^	8.2 (5.8, 10.2)^#^	4.7 (2.7, 6.4)
CA19-9 (*μ*/ml, median, range)	198.6 (31–530)^#^∗	4.2 (2.7, 10.2)^#^	2.1 (1.3, 6.9)

Notes: ^#^compared with the normal, *P* < 0.01; ∗ compared with the DM, *P* < 0.01.

**Table 2:** Relation of shared common metabolites to risk of future diseases (diabetes mellitus and pancreatic cancer).

Model	LysoPC (16 : 0)	Catelaidic acid	LysoPC (22 : 6)	Docosanamide	Cerebronic acid	Asparaginyl-histidine
*Logistic regression between diabetes mellitus patients and healthy volunteers*						
Metabolite as a continuous variable						
Per SD	1.381 (1.163–1.930)	0.137 (0.041–0.341)	1.264 (0.922–1.776)	0.271 (0.105–0.555)	0.177 (0.056–0.415)	1.845 (1.288–3.069)
*P*	0.0063	0.0002	0.1557	0.0018	0.0006	0.0051
Metabolite as a categorical variable						
1st quartile	1.0 (referent)	1.0 (referent)	1.0 (referent)	1.0 (referent)	1.0 (referent)	1.0 (referent)
2nd quartile	0.895 (0.496–1.653)	0.616 (0.217–1.312)	1.000 (0.644–1.552)	0.405 (0.144–0.822)	0.533 (0.187–1.149)	3.886 (1.966–10.953)
3rd quartile	1.759 (0.957–3.515)	0.585 (0.210–1.192)	1.063 (0.642–1.775)	0.669 (0.238–1.416)	0.526 (0.190–1.043)	3.175 (1.556–9.048)
4th quartile	3.119 (1.536–8.877)	0.232 (0.079–0.478)	1.442 (0.680–4.067)	0.306 (0.110–0.596)	0.263 (0.093–0.521)	5.241 (2.328–17.749)
*P* for trend	0.0001	<0.0001	0.8107	0.0005	0.0002	<0.0001

*Logistic regression between pancreatic cancer patients and healthy volunteers*						
Metabolite as a continuous variable						
Per SD	1.147 (1.015–1.364)	0.107 (0.028–0.291)	4.026 (2.079–11.805)	0.126 (0.032–0.330)	0.058 (0.009–0.210)	2.052 (1.386–3.556)
*P*	0.0559	0.0002	0.0011	0.0004	0.0003	0.0022
Metabolite as a categorical variable						
1st quartile	1.0 (referent)	1.0 (referent)	1.0 (referent)	1.0 (referent)	1.0 (referent)	1.0 (referent)
2nd quartile	0.493 (0.248–0.853)	0.693 (0.246–1.472)	1.000 (0.329–3.036)	0.550 (0.200–1.079)	0.771 (0.274–1.635)	2.371 (1.250–5.114)
3rd quartile	1.326 (0.781–2.390)	0.579 (0.207–1.191)	2.433 (1.232–6.771)	0.638 (0.225–1.364)	0.531 (0.190–1.080)	2.988 (1.652–6.307)
4th quartile	1.671 (0.841–4.628)	0.191 (0.056–0.430)	5.241 (2.328–17.749)	0.199 (0.059–0.450)	0.203 (0.060–0.459)	4.160 (2.012–12.181)
*P* for trend	0.0010	<0.0001	<0.0001	<0.0001	<0.0001	<0.0001

*Logistic regression between pancreatic cancer patients and diabetes mellitus patients*						
Metabolite as a continuous variable						
Per SD	0.917 (0.844–0.989)	0.807 (0.436–1.453)	2.296 (1.547–3.921)	0.775 (0.458–1.276)	0.851 (0.468–1.517)	1.116 (0.860–1.464)
*P*	0.0287	0.4781	0.0004	0.3234	0.5857	0.4117
Metabolite as a categorical variable						
1st quartile	1.0 (referent)	1.0 (referent)	1.0 (referent)	1.0 (referent)	1.0 (referent)	1.0 (referent)
2nd quartile	0.550 (0.250–1.089)	1.126 (0.722–1.775)	1.000 (0.329–3.036)	1.357 (0.838–2.289)	1.446 (0.919–2.355)	0.610 (0.209–1.428)
3rd quartile	0.754 (0.418–1.280)	0.991 (0.627–1.562)	2.289 (1.170–6.334)	0.953 (0.606–1.490)	1.010 (0.629–1.614)	0.941 (0.325–2.202)
4th quartile	0.536 (0.296–0.896)	0.822 (0.286–1.894)	3.634 (1.824–10.275)	0.652 (0.234–1.326)	0.771 (0.274–1.635)	0.794 (0.276–1.833)
*P* for trend	0.0824	0.8753	<0.0001	0.2421	0.2513	0.3034

Values are odds ratios (95% confidence intervals) for diabetes mellitus and pancreatic cancer, from logistic regressions.

**Table 3:** Relation of specific metabolites to risk of future diseases (diabetes mellitus and pancreatic cancer).

Model	Behenic acid	Nonadecanetriol	LysoPC (20 : 3)	LysoPC (16 : 1)
*Logistic regression between diabetes mellitus patients and healthy volunteers*				
Metabolite as a continuous variable				
Per SD	0.239 (0.085–0.530)	2.099 (1.237–4.506)	1.170 (0.770–1.836)	1.286 (1.080–1.749)
*P*	0.0019	0.0288	0.4690	0.0337
1st quartile	1.0 (referent)	1.0 (referent)	1.0 (referent)	1.0 (referent)
2nd quartile	0.693 (0.228–1.366)	1.401 (0.858–2.382)	0.756 (0.454–1.226)	0.956 (0.572–1.595)
3rd quartile	0.543 (0.196–1.080)	1.765 (1.038–3.191)	1.012 (0.622–1.652)	1.000 (0.594–1.683)
4th quartile	0.355 (0.128–0.689)	2.680 (1.340–7.524)	1.573 (0.780–4.376)	1.158 (0.822–3.130)
*P* for trend	0.0052	0.0219	0.2321	0.4422

*Logistic regression between pancreatic cancer patients and healthy volunteers*				
Metabolite as a continuous variable				
Per SD	0.049 (0.006–0.201)	2.915 (1.580–6.605)	2.441 (1.473–4.737)	1.017 (0.938–1.105)
*P*	0.0006	0.0029	0.0024	0.6738
1st quartile	1.0 (referent)	1.0 (referent)	1.0 (referent)	1.0 (referent)
2nd quartile	0.644 (0.228–1.366)	1.010 (0.547–1.819)	0.638 (0.314–1.153)	0.655 (0.356–1.130)
3rd quartile	0.543 (0.196–1.081)	1.688 (0.984–3.067)	0.991 (0.574–1.705)	0.812 (0.474–1.361)
4th quartile	0.193 (0.057–0.433)	3.208 (1.644–8.927)	2.475 (1.273–6.832)	1.554 (0.868–3.132)
*P* for trend	<0.0001	0.0006	0.0007	0.0348

*Logistic regression between pancreatic cancer patients and diabetes mellitus patients*				
Metabolite as a continuous variable				
Per SD	0.746 (0.403–1.317)	1.489 (0.998–2.340)	1.683 (1.175–2.596)	0.878 (0.759–0.981)
*P*	0.3230	0.0631	0.0090	0.0384
1st quartile	1.0 (referent)	1.0 (referent)	1.0 (referent)	1.0 (referent)
2nd quartile	0.928 (0.588–1.459)	0.721 (0.367–1.357)	0.843 (0.411–1.574)	0.685 (0.370–1.192)
3rd quartile	1.022 (0.637–1.648)	0.956 (0.533–1.713)	0.979 (0.583–1.632)	0.812 (0.474–1.361)
4th quartile	0.543 (0.196–1.081)	1.197 (0.680–2.121)	1.574 (0.981–2.626)	1.023 (0.631–1.658)
*P* for trend	0.3312	0.2912	0.0981	0.4267

Values are odds ratios (95% confidence intervals) for diabetes mellitus and pancreatic cancer, from logistic regressions.

**Table 4:** Test performance characteristics for the biomarker signature from 5 repeated 5-fold cross-validation.

Model	AUC (95% CI)	Sensitivity (95% CI)	Specificity (95% CI)	Accuracy (95% CI)	Kappa (95% CI)
*Logistic regression between diabetes mellitus patients and healthy volunteers*					
Combination1	0.872 (0.847–0.896)	0.760 (0.690–0.830)	0.816 (0.778–0.854)	0.779 (0.751–0.807)	0.556 (0.499–0.613)
Combination2	0.953 (0.937–0.969)	0.876 (0.839–0.913)	0.876 (0.825–0.927)	0.858 (0.828–0.889)	0.713 (0.651–0.775)
Combination3	0.870 (0.842–0.898)	0.764 (0.712–0.816)	0.807 (0.761–0.852)	0.773 (0.734–0.811)	0.538 (0.460–0.615)
Combination4	0.847 (0.810–0.884)	0.768 (0.696–0.840)	0.735 (0.687–0.782)	0.767 (0.732–0.803)	0.537 (0.468–0.607)
Combination5	0.904 (0.870–0.938)	0.830 (0.782–0.878)	0.727 (0.675–0.778)	0.792 (0.753–0.830)	0.585 (0.510–0.660)

*Logistic regression between pancreatic cancer patients and healthy volunteers*					
Combination1	0.882 (0.846–0.918)	0.816 (0.760–0.872)	0.873 (0.834–0.912)	0.852 (0.821–0.883)	0.701 (0.640–0.763)
Combination2	0.974 (0.958–0.991)	0.890 (0.847–0.933)	0.906 (0.861–0.951)	0.886 (0.864–0.909)	0.769 (0.722–0.817)
Combination3	0.879 (0.848–0.909)	0.834 (0.781–0.887)	0.896 (0.860–0.932)	0.865 (0.843–0.888)	0.728 (0.683–0.774)
Combination4	0.860 (0.823–0.896)	0.848 (0.785–0.911)	0.831 (0.778–0.883)	0.837 (0.801–0.874)	0.677 (0.605–0.750)
Combination5	0.919 (0.887–0.952)	0.894 (0.853–0.935)	0.773 (0.714–0.832)	0.841 (0.808–0.874)	0.684 (0.619–0.750)

*Logistic regression between pancreatic cancer patients and diabetes mellitus patients*					
Combination1	0.586 (0.534–0.638)	0.503 (0.439–0.566)	0.629 (0.575–0.683)	0.586 (0.556–0.615)	0.183 (0.127–0.239)
Combination2	0.631 (0.580–0.682)	0.547 (0.493–0.600)	0.613 (0.544–0.683)	0.547 (0.522–0.572)	0.100 (0.049–0.151)
Combination3	0.569 (0.516–0.622)	0.463 (0.398–0.527)	0.612 (0.543–0.681)	0.547 (0.516–0.579)	0.101 (0.038–0.165)
Combination4	0.723 (0.691–0.754)	0.635 (0.589–0.681)	0.696 (0.641–0.751)	0.677 (0.641–0.713)	0.350 (0.278–0.422)
Combination5	0.680 (0.632–0.729)	0.589 (0.535–0.643)	0.671 (0.609–0.732)	0.659 (0.626–0.692)	0.313 (0.246–0.381)

AUC, area under the curve; combination1, model with top 5 metabolites selected from one-way ANOVA test; combination2, model with top 5 metabolites selected from Tukey's honest significance test between diabetes mellitus patients and healthy volunteers; combination3, model with top 5 metabolites selected from Tukey's honest significance test between pancreatic cancer patients and healthy volunteers; combination4, model with top 5 metabolites selected from Tukey's honest significance test between pancreatic cancer patients and diabetes mellitus patients; and combination5, model with all the metabolites involved in previous 4 models.

**Table 5:** The abilities of combination2 and CA19-9 in PC diagnosis.

Project	AUC (95% CI)	Sensitivity (95% CI)	Specificity (95% CI)	Accuracy (95% CI)	Kappa (95% CI)
Combination2	0.974 (0.958–0.991)	0.890 (0.847–0.933)	0.906 (0.861–0.951)	0.886 (0.864–0.909)	0.769 (0.722–0.817)
CA19-9	0.821 (0.765–0.874)	0.791 (0.745–0.826)	0.826 (0.765–0.894)	0.813 (0.778–0.834)	0.673 (0.603–0.747)

## Data Availability

The data used to support the findings of this study are all included within the article.
